# A probabilistic assessment of the likelihood of vegetation drought under varying climate conditions across China

**DOI:** 10.1038/srep35105

**Published:** 2016-10-07

**Authors:** Zhiyong Liu, Chao Li, Ping Zhou, Xiuzhi Chen

**Affiliations:** 1 Institute of Geography, Heidelberg University, Heidelberg 69120, Germany; 2 Department of Global Ecology, Carnegie Institution for Science, Stanford, California, USA; 3 Guangzhou Institute of Geography, Guangzhou 510070, China; 4 South China Botanical Garden, Chinese Academy of Sciences, Guangzhou 510650, China

## Abstract

Climate change significantly impacts the vegetation growth and terrestrial ecosystems. Using satellite remote sensing observations, here we focus on investigating vegetation dynamics and the likelihood of vegetation-related drought under varying climate conditions across China. We first compare temporal trends of Normalized Difference Vegetation Index (NDVI) and climatic variables over China. We find that in fact there is no significant change in vegetation over the cold regions where warming is significant. Then, we propose a joint probability model to estimate the likelihood of vegetation-related drought conditioned on different precipitation/temperature scenarios in growing season across China. To the best of our knowledge, this study is the first to examine the vegetation-related drought risk over China from a perspective based on joint probability. Our results demonstrate risk patterns of vegetation-related drought under both low and high precipitation/temperature conditions. We further identify the variations in vegetation-related drought risk under different climate conditions and the sensitivity of drought risk to climate variability. These findings provide insights for decision makers to evaluate drought risk and vegetation-related develop drought mitigation strategies over China in a warming world. The proposed methodology also has a great potential to be applied for vegetation-related drought risk assessment in other regions worldwide.

Vegetation dynamics are significantly impacted by climate change around the globe. Climate controls the long-term dynamics and broad-scale distributions of vegetation. Conversely, vegetation also regulates climate through impacting energy, water, and other bio-physical processes^1^,^2^,^3^,^4^,^5^. Accordingly, investigating the response of vegetation to climate variations is critical for understanding the dynamics of terrestrial ecosystems^6^,^7^. Over the last few decades, the Normalized Difference Vegetation Index (NDVI) has been extensively used as a proxy indicator of terrestrial vegetation productivity, and has been widely applied to detect vegetation responses to climate change^8^,^9^,^10^,^11^. Moreover, NDVI provides temporally and spatially continuous information over vegetated surfaces and can thus be used for vegetation-related drought monitoring and impact assessment^12^,^13^. Comparing to traditional ground-based drought indicators including agricultural, meteorological, and hydrological ones, the satellite-based vegetation observations are able to assess the impacts of drought on ecosystem (e.g., vegetation health and growth), and produce global, near-real-time and consistent data records with improved spatial resolution^14^. NDVI (or its derivatives), as the most frequently used vegetation index, has been widely employed for quantitative monitoring and assessment of vegetation-related drought^13^,^15^.

In China, a number of studies have also explored the linkages between vegetation and climatic variables (e.g., precipitation and temperature) in various geographic regions based on a variety of datasets and statistical approaches (e.g., using correlation coefficient, linear regression models, or other relevant measures)^16^,^17^,^18^,^19^,^20^,^21^,^22^. These studies provided valuable knowledge about vegetation dynamics and the feedbacks to climate change in different parts of China. However, little effort has been made to identify the likelihood of vegetation-related drought and the sensitivity to different climate conditions across China, particularly from a perspective based on joint probability dependence. Such a probabilistic identification may offer important additional insights into the vegetation response to climate variability.

The overall objective of our study is to investigate the vegetation dynamics and the likelihood of vegetation-related drought under varying climate conditions across China using satellite remote sensing observations. First, this study examines the temporal dynamics of NDVI and climatic variables (i.e., precipitation and temperature). Then, we focus on modeling the joint probability distribution between vegetation and climatic variables based on copula theory. This joint probability distribution enables the quantification of the likelihood of vegetation-related drought and its sensitivity to different climate scenarios. Our analysis is conducted across the whole of China. Thus, the spatial patterns of vegetation related drought likelihood over China are characterized.

## Results

### Temporal dynamics of NDVI vegetation and climatic variables

**Figure 1:**
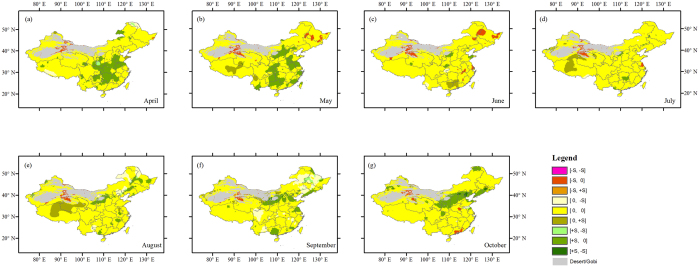
Comparison of trends of NDVI and precipitation for April–October (**a**–**g**) (note that the maps are further interpolated from the maps with a spatial resolution of 0.5° × 0.5°). Statistically significant (the absolute value of MK test equal to or greater than 1.96) positive trends are denoted as + S, negative trends as −S and insignificant ones as 0. The first character in each pair right the color bar denotes NDVI trend and the second one denotes precipitation trend. This figure was generated using ArcGIS 10.1 (http://www.esri.com/software/arcgis/).

**Figure 2:**
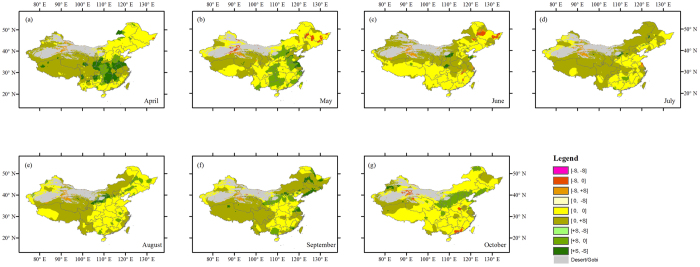
Comparison of trends of NDVI and temperature for April–October (**a**–**g**). Statistically significant (the absolute value of MK test equal to or greater than 1.96) positive trends are denoted as + S, negative trends as −S and insignificant ones as 0. The first character in each pair right the color bar denotes NDVI trend and the second one denotes temperature trend. This figure was generated using ArcGIS 10.1 (http://www.esri.com/software/arcgis/).

For each month of growing season (April–October) at each grid cell, we first examined the temporal trends of NDVI based on the MK test. We classified the NDVI trends into three categories according to the estimates of MK trend, i.e., nonsignificant, statistically significantly positive and negative trends (at the 5% significance level). We found that trends in NDVI are non-significant in growing season over most regions (Figs 1 and 2). In April, May, September, and October, significant greening is prominently seen in North China Plain and southern China. From June to August, there are fragmented patterns of significant greening, but the ratio of the lands with significant increase in NDVI is much lower. There are only a few scattered segments where NDVI exhibits a significantly negative trend, mainly in Northwest and Northeast China.

Since climatic variables such as temperature and precipitation are the key elements limiting vegetation growth. In light of this, we compared trends in NDVI with those in both precipitation and temperature for each month of growing season at each grid cell based on the spatially matching changes in NDVI and climatic variables. As shown in Fig. 1, the trends of NDVI and precipitation are of the same sign with non-significant changes for most areas of the country. In fact, we found the areas (mainly in North China Plain and southern China) with significant increase in NDVI mostly show non-significant changes in precipitation. Analysis regarding NDVI-temperature changes reveals that most of the areas with significant increase in NDVI generally correspond to non-significant temperature variability for most months (Fig. 2). Surprisingly, no significant changes in NDVI is found for the cold regions such as Northwest China, Inner Mongolia, and Qinghai-Tibet Plateau where warming is significantly increasing based on the MK trend test, although temperature is generally a very important factor governing the vegetation growth in such regions.

### Illustration of the copula-based approach

In addition to the linkage between NDVI and temperature/precipitation trends as presented above, one (e.g., the decision-makers) might also be interested in quantitative estimates of the likelihood of vegetation-related droughts under dynamic climate conditions. As an example, we demonstrate here the use of the copula-based joint probability distribution in quantifying the likelihood of vegetation-related drought under given precipitation (or temperature) conditions on an arbitrarily selected a pixel (115°27′19.6″E 43°47′26.993″N) in Xilin Gol Grassland, Inner Mongolia. We focus on NDVI and precipitation (or precipitation) observations over April–October (the growing season). For each month, the appropriate marginal distributions of NDVI and precipitation (or temperature) were first determined. Then, their joint probability distributions were constructed using the proposed copula-based method by joining the marginal distributions. Once the joint probability distribution was determined, the conditional distribution of NDVI given precipitation or temperature can be obtained using equation (2).

**Figure 3:**
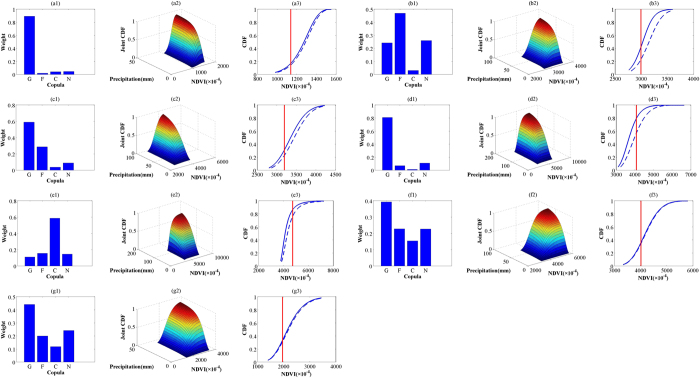
Weight values of four copulas (left) (G, F, C, and N indicates Gumbel, Frank, Clayton, and Normal, respectively), joint CDF using the best-fitted copula (middle), and conditional CDF of NDVI under two precipitation scenarios: *u*_*P*_ ≤ 20% (blue solid curves) and *u*_*P*_ ≤ 60%(blue dashed curves), with the specific NDVI drought threshold (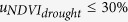) shown as red lines (right), for April–October (**a**–**g**).

Table 1 presents the Kolmogorov–Smirnov statistics of different theoretical distributions for NDVI and precipitation in different months. Figure 3 shows the weights of different copula functions, the joint CDF using the best-fitted copula, and the conditional distribution of NDVI under two precipitation scenarios (*u*_*P*_ ≤ 20% and *u*_*P*_ ≤ 60%) for each month (see Fig. S1 with corresponding results for temperature, and Figs S2 and S3 for precipitation and temperature at the other selected pixel located in eastern Hunan province (as a representative of humid regions), respectively). Note that the best-fitted copula may vary from month to month, indicating the seasonality of the interaction between NDVI and precipitation as well as the importance of selecting the best copula function from a set of candidates. The conditional distribution of NDVI for the two precipitation scenarios suggests that increase in precipitation will produce a positive effect of varying magnitude on vegetation greening during the entire growing season, although the effect is very weak in September and October (the probability curves for the two given scenarios become close together). In addition, Fig. 3 also shows the vegetation-related drought probability (using a NDVI threshold of 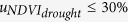) under the two precipitation levels (right, red lines). This value is interpreted as the likelihood of vegetation-related drought under a given precipitation level, with smaller values implying a lower likelihood of drought.

### Spatial patterns of the dependence between NDVI and precipitation

For each grid cell across China, we examined the spatial patterns of the probability of a given vegetation-related drought (i.e., the probability of 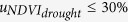) conditioned on two precipitation scenarios, denoted as *u*_*P*_ ≤ 20% and *u*_*P*_ ≤ 60%, respectively. This allows us to identify the most likely regions of drought, and determine which regions are sensitive to precipitation during the growing season. It should be emphasized here that one is able to investigate the drought risk and delineate the corresponding risk maps across China for any self-defined NDVI drought thresholds, precipitation or temperature scenarios of interest (not limited to the cases in the current study), once the copula-based joint probability distribution has been constructed.

**Figure 4:**
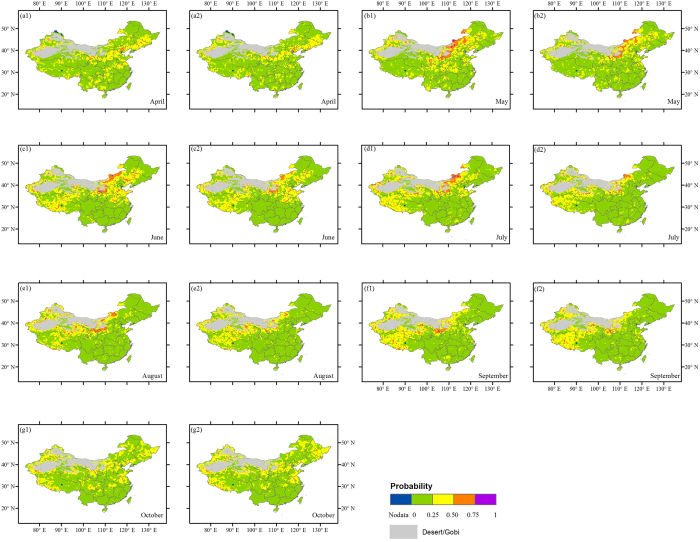
Drought probability (risk) of vegetation for the particular NDVI threshold (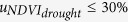) under *u*_*P*_ ≤ 20% (1) and *u*_*P*_ ≤ 60% (2) precipitation scenarios across China from April–October (**a**–**g**). The background is an administrative map of China. This figure was generated using ArcGIS 10.1 (http://www.esri.com/software/arcgis/).

Figure 4 maps the probability of vegetation-related drought under the two precipitation scenarios from April to October. Over April–August, scattered grid spots with high likelihood (>50%) of drought under the low-precipitation level are mostly concentrated over Inner Mongolia, Northwest China, and Qinghai-Tibet Plateau (with an arid or semi-arid climate) (Fig. 4a–f). The increase in precipitation has a positive effect on reducing drought risk to a lower level (e.g., lower than 50%) at a few of these spots. However, the effect is unapparent for most of them which still experience a high drought likelihood even when precipitation is at a relatively high level (i.e., *u*_p_ < 60%). In October, few isolated spots with high likelihood of drought are found (Fig. 4g). Additionally, under both given precipitation scenarios, the drought likelihood is relatively low (lower than 25%) across most of southern China, which corresponds to a main humid region of the country. Particular attention should be paid to a few pixels where the drought probability increases as precipitation increases during the growing season, e.g., some pixels in Northeast China in October (Fig. 4g2), and in South China in April (Fig. 4a2).

### Spatial patterns of the dependence between NDVI and temperature

**Figure 5:**
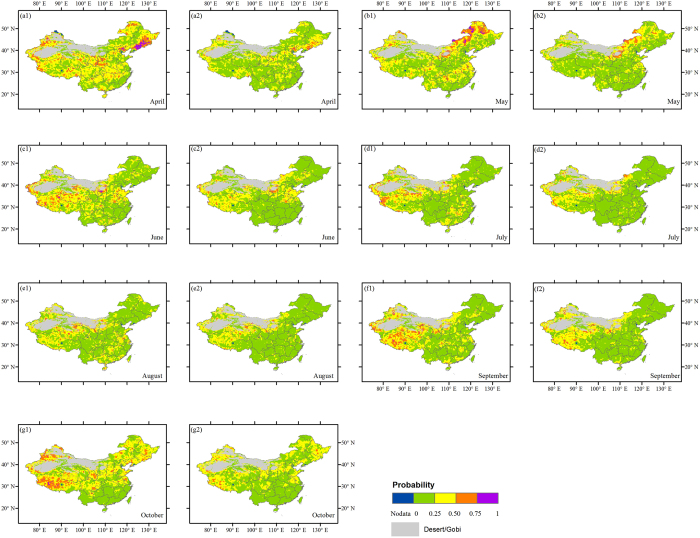
Drought probability (risk) of vegetation for the particular NDVI threshold (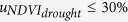) under *u*_*T*_ ≤ 20% (1) and *u*_*T*_ ≤ 60% (2) temperature scenarios across China from April to October (**a**–**g**). The background is an administrative map of China. This figure was generated using ArcGIS 10.1 (http://www.esri.com/software/arcgis/).

Figure 5 presents the spatial patterns of the likelihood of a given vegetation-related drought (i.e., the probability of 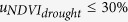) conditioned on two temperature scenarios for each month in the growing season. Over April–October, the low-temperature condition is related to a high likelihood (>50%) of vegetation-related drought in some parts of China (Fig. 5a–g), e.g., in Northeast China (in April, May and October), Northwest China (in April–October), Qinghai-Tibet Plateau (in April–October), North China Plain (in April, June and October), and several scatter locations in southern China for some months. In general, shifting to the higher-temperature condition (Fig. 5) remarkably reduces the likelihood of drought. This implies that temperature plays a positive role in vegetation growth over most areas of China during the growing season, including these cold regions. It should, however, be noted that higher temperature may also become a limiting factor for vegetation growth in a few spots, e.g., some scatter grid cells in the central Inner Mongolia in June–August (Fig. 5c–e).

### Vegetation drought stress analysis

Since a certain NDVI drought threshold (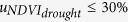) was considered in the above analysis, we further investigated the vegetation-related drought risk with other drought stress level. In this section, the 20th percentile of NDVI (denoted as 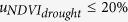) was considered as the NDVI drought threshold. Following the same approach, we examined the spatial patterns of drought likelihood with this given NDVI drought threshold. Also, it should be mentioned here that any NDVI drought threshold of interest could be detected based on the established joint dependence structure between NDVI and climatic variables. For the severer drought threshold, not surprisingly, the drought likelihood has decreased to some extent for each month (Figs S4–S5 vs. Figs 4 and 5). This confirms that varying NDVI drought thresholds can modify the corresponding drought likelihood for the given climate conditions although the changes in risk are not consistent with different cases (months). Moreover, it can be found that a higher threshold (e.g., 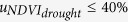) generally produces increasing drought likelihood and more regions with the likelihood over 50% as compared to lower thresholds (results not shown).

## Discussion

Despite increasing concerns about the response of vegetation to climate change, investigation of the likelihood of vegetation-based drought under different climate conditions is still remarkably scarce, particularly at China-wide scale. In this study, we first compared the temporal trends of vegetation (NDVI) and climatic variables (precipitation and temperature) in growing season (April–October) over China using the nonparametric MK trend test. Although trends in vegetation over China have been investigated in previous studies using linear regression models^17^,^23^, our analyses based on a nonparametric trend detection technique provide additional information about the trend in vegetation over China. In particular, our results show that most regions across China do not exhibit significant trends in NDVI in growing season. The significant greening regions are mainly distributed in North China Plain and southern China for most months of growing season, generally corresponding to a non-significant trend in precipitation. No significant changes in NDVI are found for the most parts of cold regions such as Northwest China, Inner Mongolia, and Qinghai-Tibet Plateau where warming is significantly increasing. A few scattered spots in Northwest China exhibit a significant decrease in NDVI corresponding to a non-significant trend in precipitation and significant warming trend, indicating that the temperature might dominantly impact the temporal dynamics of vegetation over these spots. It can also be noticed that there are significant increasing trends in NDVI over northern China in October while non-significant significant trends are found in both temperature and precipitation. This could be partially explained by the nonlinearity of vegetation responses to climate change. Moreover, it is unlikely that climate drivers alone determine the vegetation dynamics^21^. Some other possible factors such as changes in soil moisture conditions, atmospheric CO_2_ concentration, nitrogen deposition, as well as agricultural and land use polices (e.g., afforestation) may also strongly affect the vegetation variations^3^,^24^.

Then, we presented a copula-based bivariate probabilistic model to identify the joint dependence between NDVI and climatic variables (precipitation and temperature). This enabled us to develop the assessment of vegetation-related drought likelihood conditioned upon different precipitation or temperature levels for the growing season across China over the period 1982–2012. To the best of our knowledge, our study is the first to evaluate the impacts of global warming-induced changes in precipitation and temperature on vegetation-related drought from a probabilistic perspective. The proposed approach was expanded to characterize the spatial patterns of conditional drought probability associated with vegetation across the whole of China. The areas most likely to suffer drought and those sensitive to precipitation or temperature were thus identified from the risk maps.

Our results indicate that the spatial patterns of vegetation-related drought risk under scarce precipitation condition vary markedly across the growing season. The lands with a high likelihood of vegetation-related drought (i.e., greater than 50%) are dominantly situated in arid and semiarid zones (e.g., Inner Mongolia, Northwest China and Qinghai-Tibet Plateau) which are vulnerable to extreme conditions. The increase of precipitation has limited effect on reducing the vegetation drought risk to the level of lower than 50% for most of these lands. A possible hypothesis for the insensitivity to precipitation is that the plants in these regions are exposed to sustained water deficits and may have physiological mechanisms to tolerate these situations.

We also found low likelihood of vegetation-related drought in southern China (subhumid and humid regions) even under the relatively low precipitation level. Vegetation (e.g., forests) in these regions is generally easier to access to deeper groundwater as compared to arid and semiarid lands (vegetation mainly characterized by grasslands) and have a positive water balance. It might be concluded that the plants (e.g., forests) in humid areas have a higher resilience to extreme fluctuations in precipitation than the grasslands in arid areas. However, some studies pointed out that forests could be less resilient to extreme events than grasslands (rapid to recover from extreme disturbance) when their mortality thresholds are reached^25^,^26^. This current study did not quantify such thresholds for different vegetation types across China under extreme climate events. A quantitative and systematic assessment would be needed in the further research. Additionally, we found a few spots over China exhibiting negative NDVI-precipitation dependence, which suggests that the available moisture conditions in these spots are sufficient and close to saturation for vegetation growth. This means that any increase in precipitation may not be beneficial to plant growth, and could even prohibit growth^27^. Moreover, precipitation may increase the level of cloud cover. This will result in a reduction of incident radiation, which will hinder photosynthesis in vegetation^28^.

From the analyses under different temperature conditions, we also noticed that spatial variability of vegetation-related drought likelihood is large among different months. Nevertheless, it is possible to identify some general patterns. For instance, under low temperature scenario, the drought likelihood over 50% can also be found in some parts of Northwest China and Qinghai-Tibet Plateau. In addition to these regions, we also observed that some lands in Northeast China and North China Plain for some months exhibit similar drought likelihood. These lands are the main agricultural zones for China. Under high temperature condition, we found that the drought likelihood remarkably declines to a lower level in most of the original high-likelihood areas. This implies that temperature plays a relatively positive role in vegetation growth over most areas of China during the growing season. In general, higher temperature provides more adequate heat conditions, contributing to an acceleration in photosynthesis and respiration in vegetation, and this thereby benefits the vegetation growth, particularly in cold regions (e.g., Qinghai-Tibet Plateau and the northern part of China)^29^,^30^. We also found the temperature generally shows a significant increasing trend (Fig. 2) in these cold regions. This may be beneficial for vegetation activity and reducing the potential of vegetation-related drought. However, we did not know whether the significant changes in temperature over these regions will be beyond the capacity of vegetation to adapt or recover. This may involve analyses of vegetation resilience and recovery timing after temperature extremes, particularly for different vegetation types. Although our study did no focus on these topics, the vegetation-related drought likelihood under different climate conditions also provides promising information for constituting such analyses.

The current study mainly focuses on the impacts of precipitation and temperature on vegetation-related drought separately, based on a bivariate conditional model. However, extreme climate events could be concurrent^31^, e.g., concurrent extreme low precipitation and high temperature events. In light of this, we further investigated the vegetation-related drought risk under concurrent extreme climate events across China. As an illustration, we considered the concurrent extreme low precipitation (the 20th percentiles of precipitation) and high temperature (the 60th percentiles of temperature) scenarios. Therefore, we modeled the trivariate conditional distributions for the three variables (i.e., NDVI, precipitation, and temperature) by using 3-dimensional vine copulas (see Equation (S1) in supplementary information). Figure S6 (supplementary information) demonstrates the risk maps of vegetation-related drought under the given concurrent extreme climate scenarios (i.e., the 20th percentiles of precipitation and the 60th percentiles of temperature) across China from April to October. Due to the estimation based on the trivariate joint dependence of NDVI, precipitation, and temperature, it is clear that vegetation-related drought under the given concurrent extreme climate scenarios exhibits different spatial risk patterns when compared to the analyses based on the bivariate joint dependence of NDVI-precipitation or NDVI-temperature (Figs 4 and 5). Under the joint effect of the given concurrent low precipitation and high temperature (having positive impact on vegetation according to Fig. 5) events, it can be seen that there are few grid spots with the likelihood of drought over 50% for most months (except August). Lands with the drought likelihood over 25% are mostly found in northern and western parts of China for most months, and also observed in southern China for certain months (particularly in June and October). Despite only specific concurrent extreme climate scenarios analyzed in the current study, it should be emphasized that any concurrent extreme climate scenarios of interest could be examined as soon as the trivariate joint dependence of NDVI, precipitation and temperature has been established.

It is noteworthy that the relationships between vegetation and climatic variables and the corresponding vegetation-related drought may also be strongly affected by human disturbances. For instance, croplands are planted and harvested based on an annual plan. The response of croplands on climate variability may be disturbed by the management actions taken (e.g., irrigation, replanting of a failed crop, or longer term adaptation)^26^,^32^,^33^. Other human disturbances in China such as afforestation (e.g., the three-north shelter forest program and other similar project in other provinces) strongly affect the vegetation trend and the drought risk assessment under climate change^3^,^34^. Desertification also has an impact on the vegetation variations and the related drought. In northern China, desertification is not only related to the severe climate conditions, but also the short-term and long-term unsustainable human activities such as extensive deforestation and over-reclamation^35^. In addition, human disturbances like over-grazing exert a significant impact on the grassland degradation. Some existing studies reported that the human activities in past decades were directly responsible for the grassland degradation and the vegetation variations in the Three-River Headwaters Region^36^. Overall, the presented probabilistic assessment of vegetation and climatic variables may improve our understanding of vegetation shifts in a warming world and provides important additional information for the development of drought mitigation strategies (e.g., the land areas vulnerable to vegetation-related drought or sensitive to climate change) as compared to the deterministic approaches. It may also contribute to the monitoring and prediction of vegetation-related drought events under future climate conditions, and will serve as an important component in a national drought monitoring and assessment network.

It should be pointed out that data limitations (particularly the limited NDVI record length) represent a serious challenge to the current methodology, as well as other NDVI-related studies. Long-term data would presumably give a better representation of the joint probability distribution of NDVI and precipitation/temperature. These would allow to more comprehensively and accurately capture interactions between vegetation and climate behaviors. Despite the relatively limited record available here, the proposed approach performs reasonably well. Another limitation is that the historic climate observations for assessing the vegetation-related drought risk were analyzed under the stationary climate assumption. The physical climatological mechanisms determining climate extremes and the impacts related to the large-scale climate drivers are not considered in the present study. For instance, climate conditions vary on a variety of time scales such as seasonal, annual, decadal, as well as multi-decadal scales^37^. Such climate dynamics on different time scales may impact the periodicities of drought risk events and the drought uncertainty. Some studies have also reported that large-scale ocean-atmospheric circulation processes such as El Niño and Southern Oscillation (ENSO), Pacific Decadal Oscillation (PDO), and Indian Ocean Dipole (IOD) have a potential influence on climate variations (e.g., climate extremes) over China and the vegetation-related drought risk^38^,^39^,^40^.

Future efforts will focus on employing a higher-dimensional copula model to identify the dependence among more meteo-hydrological variables (e.g., evapotranspiration and soil moisture) and vegetation and explore their joint impacts on vegetation. In the current study, only two climate scenarios were considered, drought likelihood under other climate scenarios may also be of importance to detect in the further work. Additionally, for the sake of simplification, in this study we used certain NDVI thresholds representing vegetation drought level. In order to better improve the vegetation-related drought monitoring and risk assessment, more works should be focused on developing combined vegetation drought indicators that involve multiple satellite datasets (bands or channels), e.g., coupling the reflective-based information and thermal-based information. Additionally, the climate-related vegetation dynamics play an important role in regulating the terrestrial carbon cycle, and the vegetation drought may have negative impacts on regional carbon balance and subsequent feedback to regional and global climate conditions^41^,^42^,^43^,^44^. A potential extension of the study in the further work is to investigate the implications of abrupt shifts in vegetation and vegetation drought to carbon cycle at China-wide scale, particularly by incorporating the terrestrial cycle models.

## Methods

### Data used

We obtained the 0.5° × 0.5° gridded monthly precipitation and temperature over China for 1982 to 2012 from the National Climatic Centre of the China Meteorological Administration (CMA) (http://cdc.cma.gov.cn/). We extracted the NDVI data over China for the same time period from the latest version of the Global Inventory Modeling and Mapping Studies (GIMMS) NDVI dataset (termed NDVI3g), which is at a spatial resolution of 8 km × 8 km, and 15-day intervals (http://ecocast.arc.nasa.gov/data/pub/gimms/3g/)^45^. This NDVI dataset was selected because it is the longest continuous satellite-derived vegetation-condition time series currently available^28^. To keep consistent with the gridded climatic data, we re-sampled the NDVI data into a spatial resolution of 0.5° × 0.5° using a nearest-neighbor assignment algorithm. We focused on the dependence between vegetation and climatic variables during the growing season, which was defined as April–October across the whole country^27^. It should be mentioned that the actual growing season may vary from one region to another.

### Mann–Kendall trend test

We used the nonparametric Mann–Kendall (MK) trend test to estimate the trend in NDVI vegetation and climate variables (i.e., precipitation and temperature) and to evaluate whether the trend is statistically significant (here, at the 5% significance level). One important advantage of this trend test is that the data need not conform to any particular distribution^46^. More details about the MK trend test can be found in some existing publications^47^.

### Probabilistic estimation model

The dependence structure of multivariate distributions can be constructed by employing classical distributions such as the multivariate normal^48^. However, the dependence between vegetation and climatic variables is usually very complex and varies in both time and space. Thus, a Gaussian method may not be appropriate for generating the dependence structure when large asymmetries exist^48^,^49^. Copulas provide an elegant means of modeling the joint dependence structure between vegetation and climatic variables. They allow the dependence to be modeled without any restriction on the marginal distribution. We therefore model the joint probability distribution of NDVI and each climatic variable (i.e., precipitation or temperature) by using copula theory. According to Sklar’s theorem^50^, the joint probability distribution of NDVI (denoted by *X*_1_) and precipitation (or temperature) (denoted by *X*_2_) *F(x*_*1*_*, x*_*2*_) can be expressed as:


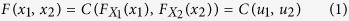


where *F*_*X*1_(*x*_1_) and *F*_*X*2_(*x*_2_) represents the marginal probability distribution of NDVI and precipitation (or temperature), respectively. *u*_*1*_ and *u*_*2*_ refer to the cumulative distribution functions (CDFs) of *x*_*1*_, *x*_*2*_. *C* is a copula function.

Before modeling the joint probability distribution, we need to fit an appropriate marginal probability distribution for each variable, i.e., NDVI and precipitation (or temperature). To determine the best fit, we compared six commonly used theoretical probability distributions including Gaussian, gamma, lognormal, Weibull, generalized Pareto, and generalized extreme value distributions. Parameters of each distribution were estimated by the maximum likelihood method. We selected the best distribution based on the Kolmogorov–Smirnov goodness-of-fit test (K-S). Once the best marginal probability distributions are determined, a proper copula function is required to model the joint probability distribution. Likewise, we considered several copula functions that have been widely used in meteo-hydrological applications, i.e., the Gumbel, Frank, Clayton and Gaussian (Normal) copulas^51^. To select the copula that best captures the dependence structure between NDVI and precipitation (or temperature), we used the Bayesian-integrated copula assessment method^52^. This method is built on a straightforward application of Bayesian analysis, and measures the performance of each copula with a corresponding weight value. It presents several advantages. For instance, it has a simple interpretation, is independent of parameter choices, easy to implement numerically, and provides reliable identification, even for small samples. According to this assessment method, the final selection is based on the copula with the highest weight. For each month considered (the vegetation growing periods) and each grid cell, there exists a 31-member (31-year dataset) data-pool of NDVI versus precipitation (or temperature) pairs, which was used to fit a joint probability distribution.

Given the joint probability distribution of NDVI and precipitation (or temperature), i.e., Equation (1), the conditional probability distribution of NDVI under a given precipitation or temperature scenario can be derived. In practice, one might be interested in the specific conditional probabilities of *X*_*1*≤_*x*_*1*_ given *X*_*2*≤_*x*_*2*_, which can be expressed as follows^53^:


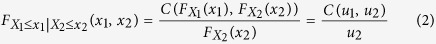


Equation (2) is applied to calculate the conditional probability distribution of NDVI under different precipitation (or temperature) conditions. In fact, an *u*_*1*_ event could be defined as “dangerous” if *u*_*1*_ exceeds (or never exceeds) a certain threshold^51^. In the current study, we simply deem vegetation to be in “drought” (i.e., the NDVI drought threshold) at a location if the corresponding NDVI value is less than the 30th percentile of NDVI over the examined period, denoted as 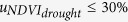. We also examined the sensitivity of vegetation drought with other NDVI thresholds (e.g., 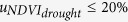). We focused on two precipitation (temperature) scenarios: the non-exceedance of the 20th and 60th percentiles of precipitation (temperature), denoted as *u*_*P*_ ≤ 20% (*u*_*T*_ ≤ 20%) and *u*_*P*_ ≤ 60% (*u*_*T*_ ≤ 20%). The percentiles of the NDVI and climatic variables were calculated from the 31-year dataset. It is noted that based on the established joint probability distribution of NDVI and precipitation (or temperature), any NDVI thresholds and precipitation/temperature scenarios of interest could be similarly determined by following Equation (2).

## Additional Information

**How to cite this article**: Liu, Z. *et al*. A probabilistic assessment of the likelihood of vegetation drought under varying climate conditions across China. *Sci. Rep*. **6**, 35105; doi: 10.1038/srep35105 (2016).

## Supplementary Material

Supplementary Information

## Figures and Tables

**Table 1:** Goodness-of-fit statistics (Kolmogorov–Smirnov statistics) of different theoretical distributions for NDVI and precipitation in different months. The best-fitted distributions are shown in bold.

Distributions	April	May	June	July	August	September	October
NDVI							
Gaussian	0.097	0.087	0.095	0.116	0.139	0.101	0.074
Gamma	0.103	**0.087**	0.089	0.096	0.128	0.094	0.061
Lognormal	0.107	0.092	0.091	0.097	0.116	**0.086**	0.062
Weibull	0.103	0.100	0.116	0.115	0.128	0.102	0.100
Generalized Pareto	0.138	0.101	0.119	0.104	**0.086**	0.129	0.079
GEV	**0.093**	0.125	**0.089**	**0.082**	0.095	0.086	**0.053**
Precipitation							
Gaussian	0.173	0.179	0.122	0.136	0.135	0.193	0.118
Gamma	**0.111**	0.121	0.103	0.078	0.097	0.126	0.129
Lognormal	0.136	0.097	0.096	0.075	0.109	**0.124**	0.190
Weibull	0.114	0.111	0.108	0.090	0.084	0.151	0.152
Generalized Pareto	0.123	0.123	0.121	**0.074**	**0.072**	0.132	0.123
GEV	0.119	**0.086**	**0.095**	0.074	0.102	0.133	**0.104**
