# Supplementary material for: A probabilistic assessment of the likelihood of vegetation drought under varying climate conditions across China
Source: Sci Rep. 2016 Oct 7;6:35105. doi: 10.1038/srep35105 (PMC5054395; doi:10.1038/srep35105)
Supplement: Supplementary Information [file srep35105-s1.pdf]

A probabilistic assessment of the likelihood of vegetation drought under varying climate conditions across China

Zhiyong Liu[1,2], Chao Li[3], Ping Zhou[4*], Xiuzhi Chen[5]

[1]*Institute of Estuarine and Coastal Research, School of Marine Sciences, Sun Yat-sen University, Guangzhou 510275, China*

[2]*Institute of Geography, Heidelberg University, Heidelberg 69120, Germany*

[3]*Department of Global Ecology, Carnegie Institution for Science, Stanford, California, USA*

[4] *Guangzhou Institute of Geography, Guangzhou 510070, China*

[5]*South China Botanical Garden, Chinese Academy of Sciences, Guangzhou 510650, China*

* Corresponding author: No. 100, the Middle of Xianlie Road, Yuexiu District, Guangzhou, 510070, China. Tel: +86 20 87033527; Fax: +86 020 87031245; E-mail: zhoupinger@gmail.com (P. Zhou)

Equation (S1):

The conditional probabilities of X_1 (NDVI) given X_2 (precipitation), X_3 (temperature) by using the high-dimensional vine copula[1] can be expressed as:

$$F(u_3 \mid u_1, u_2) = \frac{\partial C_{u_3, u_2 | u_1}(F(u_3 \mid u_1), F(u_2 \mid u_1))}{\partial F(u_2 \mid u_1)}$$

Where u_i ($i = 1, \ldots, n$) denotes the cumulative distribution functions (CDFs) of x_i .C indicates the vine copula. More details about this issue could be found in Liu et al. (2015)[2].

1. Aas, K., Czado, C., Feigessi, A., & Bakken, H. Pair-Copula Construction of Multiple Dependence. *Insurance Math. Econom.* 44 (2), 182–198 (2009), doi: 10.1016/j.insmatheco.2007.02.001.

2. Liu, Z., Zhou, P., Chen, X., & Guan, Y. A multivariate conditional model for streamflow prediction and spatial precipitation refinement. *J. Geophys. Res.: Atmos.* (2015) doi: 0.1002/2015JD02378.

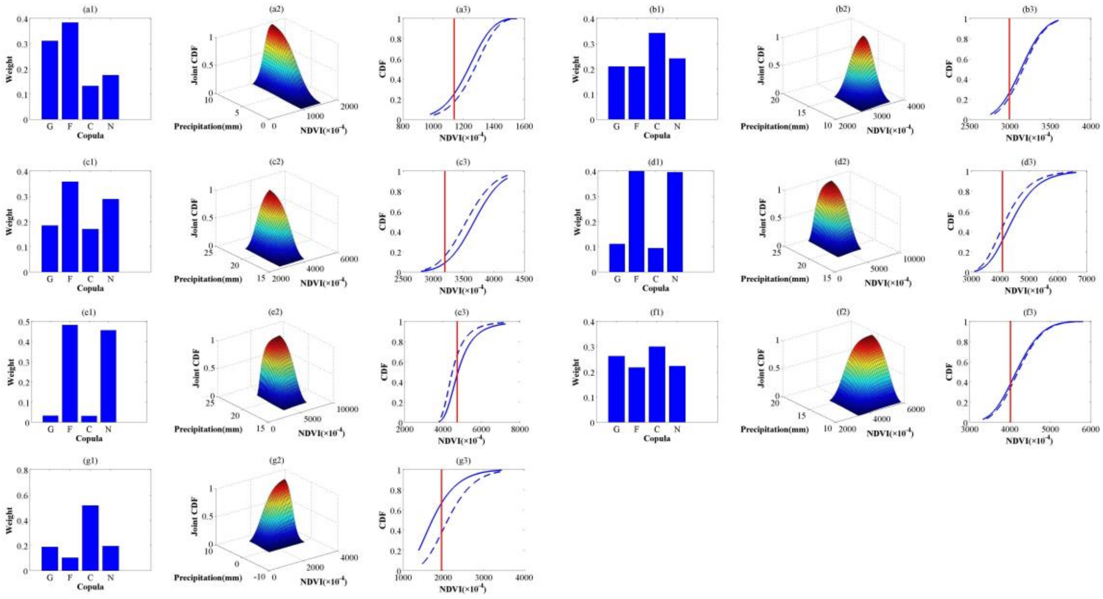

Supplementary Figure S1. Weight values of four copulas (left) (G, F, C, and N indicates Gumbel, Frank, Clayton, and Normal, respectively), joint CDF using the best-fitted copula (middle), and conditional CDF of NDVI under two temperature scenarios: $u_P \le 20\%$ (blue solid curves) and $u_P \le 60\%$ (blue dashed curves) at the selected pixel in Xilin Gol Grassland, Inner Mongolia, with the specific NDVI drought threshold ($u_{NDVI_{drought}} \le 30\%$) shown as red lines (right), for April–October (a–g).

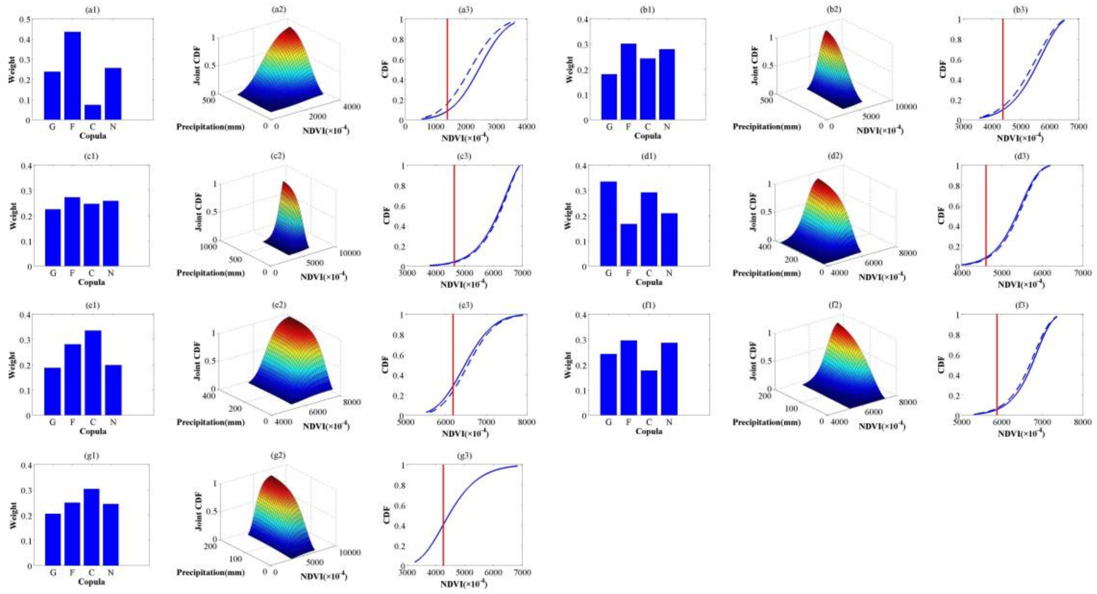

Supplementary Figure S2. Weight values of four copulas (left) (G, F, C, and N indicates Gumbel, Frank, Clayton, and Normal, respectively), joint CDF using the best-fitted copula (middle), and conditional CDF of NDVI under two precipitation scenarios: $u_P \leq 20\%$ (blue solid curves) and $u_P \leq 60\%$ (blue dashed curves) at the selected pixel in eastern Hunan province, with the specific NDVI drought threshold ($u_{NDVI_{drought}} \leq 30\%$) shown as red lines (right), for April– October (a–g).

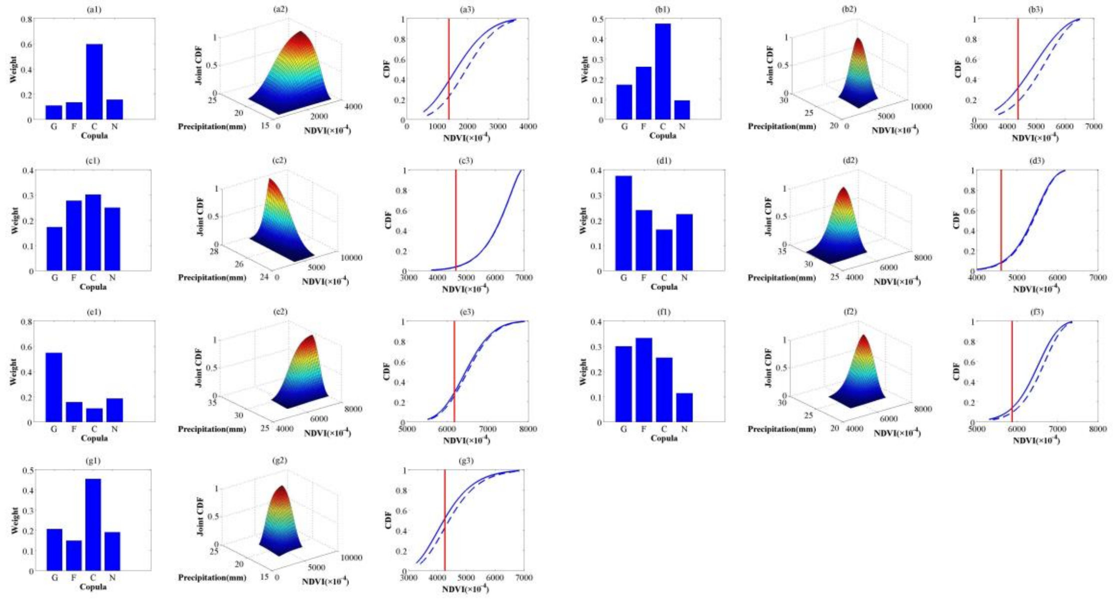

Supplementary Figure S3. Weight values of four copulas (left) (G, F, C, and N indicates Gumbel, Frank, Clayton, and Normal, respectively), joint CDF using the best-fitted copula (middle), and conditional CDF of NDVI under two temeprature scenarios: $u_P \leq 20\%$ (blue solid curves) and $u_P \leq 60\%$ (blue dashed curves)

at the selected pixel in eastern Hunan province, with the specific NDVI drought threshold ($u_{NDVI_{drought}} \leq 30\%$) shown as red lines (right), for April– October (a–g).

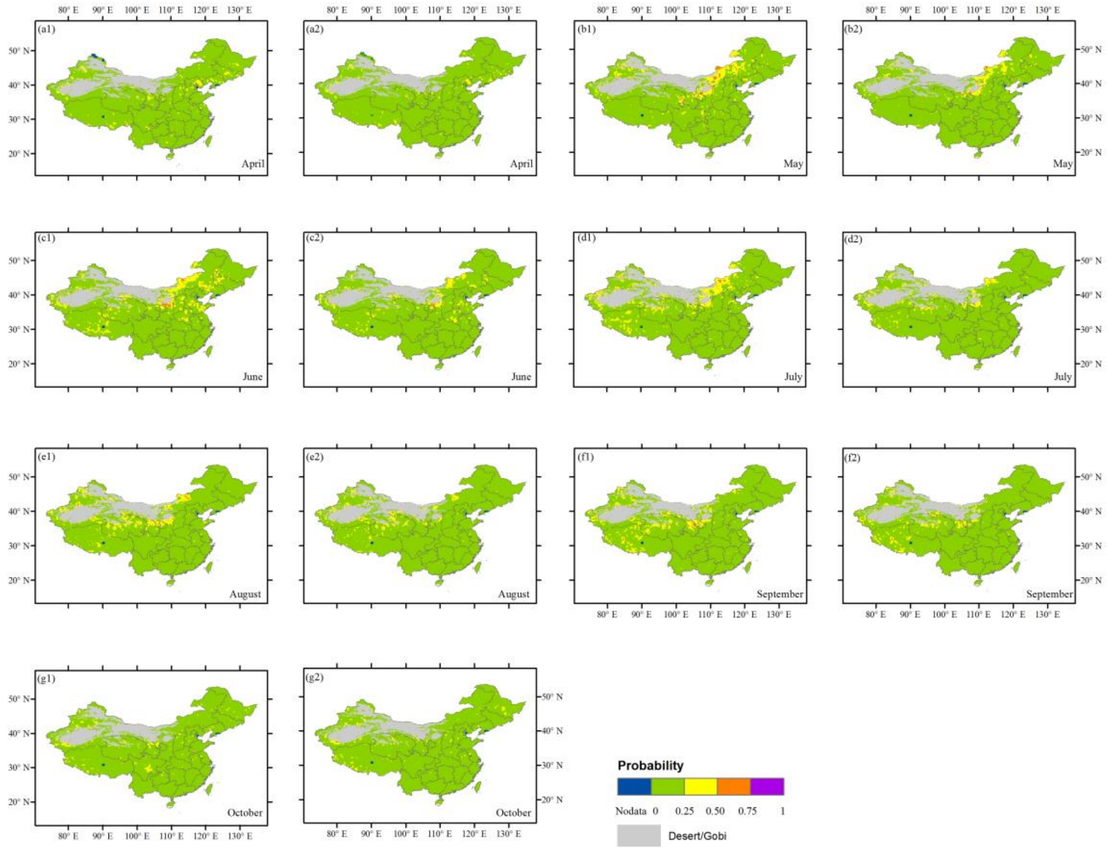

Supplementary Figure S4. Drought probability (risk) of vegetation for the particular NDVI threshold ($u_{NDVI_{drought}} \leq 20\%$) under $u_P \leq 20\%$ (left) and $u_P \leq 60\%$ (right) precipitation scenarios across China from April–October (a–g). The background is an administrative map of China. This figure was generated using ArcGIS 10.1 (http://www.esri.com/software/arcgis/).

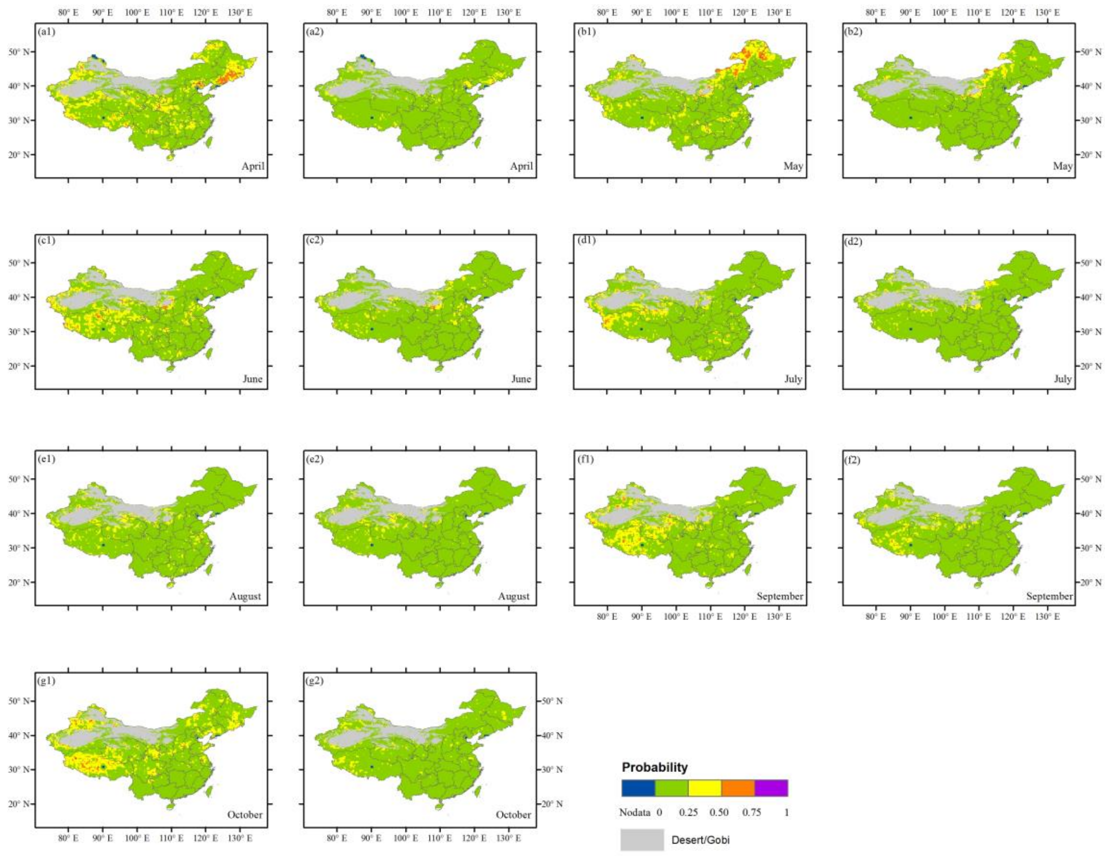

Supplementary Figure S5. Drought probability (risk) of vegetation for the particular NDVI threshold ($u_{NDVI_{drought}} \leq 30\%$) under $u_T \leq 20\%$ (left) and $u_T \leq 60\%$ (right) temperature scenarios across China from April to October (a–g). The background is an administrative map of China. This figure was generated using ArcGIS 10.1 (http://www.esri.com/software/arcgis/).

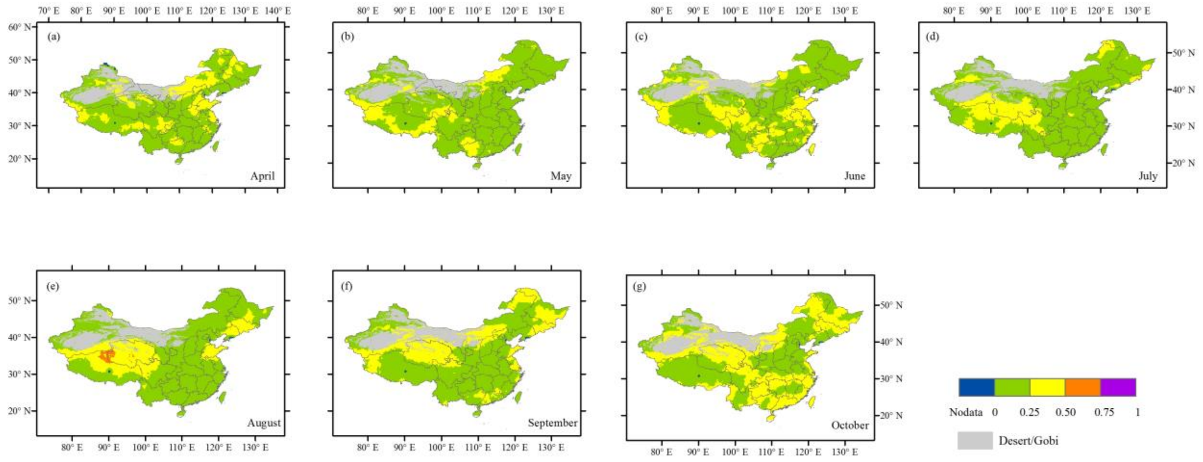

Supplementary Figure S6. Drought probability (risk) of vegetation for the particular NDVI threshold ($u_{NDVI_{drought}} \leq 30\%$) under the given concurrent extreme climate scenarios (the 20th percentiles of precipitation and the 60th percentiles of temperature) across China from April to October (a–g). The background is an administrative map of China. This figure was generated using ArcGIS 10.1 (http://www.esri.com/software/arcgis/).
